# Influenza vaccination and risk for cardiovascular events: a nationwide self-controlled case series study

**DOI:** 10.1186/s12872-020-01836-z

**Published:** 2021-01-13

**Authors:** Abhijit Sen, Inger Johanne Bakken, Ragna Elise Støre Govatsmark, Torunn Varmdal, Kaare Harald Bønaa, Kenneth Jay Mukamal, Siri Eldevik Håberg, Imre Janszky

**Affiliations:** 1 Department of Public Health and Nursing, Faculty of Medicine and Health Sciences, Norwegian University of Science and Technology, Håkon Jarls gate 11 and Mauritz Hanssens gate, 7491 Trondheim, Norway; 2 Center for Oral Health Services and Research (TkMidt), Trondheim, Norway; 3 Centre for Fertility and Health (CeFH), Norwegian Institute of Public Health, Oslo, Norway; 4 Department of Medical Quality Registries, St. Olav’s University Hospital, Trondheim, Norway; 5 Department of Circulation and Medical Imaging, Norwegian University of Science and Technology, Trondheim, Norway; 6 Clinic for Heart Disease, St. Olav’s University Hospital, Trondheim, Norway; 7 Department of Community Medicine, UiT Arctic University of Norway, Tromsø, Norway; 8 Department of Medicine, Beth Israel Deaconess Medical Center, Boston, MA USA; 9 Regional Center for Health Care Improvement, St Olav’s Hospital, Trondheim, Norway; 10 Norwegian Directorate of Health, Trondheim, Norway

**Keywords:** Influenza, Vaccination, Pandemic, Myocardial infarction, Stroke, Pulmonary embolism, Risk

## Abstract

**Background:**

US and European guidelines diverge on whether to vaccinate adults who are not at high risk for cardiovascular events against influenza. Here, we investigated the associations between influenza vaccination and risk for acute myocardial infarction, stroke and pulmonary embolism during the 2009 pandemic in Norway, when vaccination was recommended to all adults.

**Methods:**

Using national registers, we studied all vaccinated Norwegian individuals who suffered AMI, stroke, or pulmonary embolism from May 1, 2009 through September 30, 2010. We defined higher-risk individuals as those using anti-diabetic, anti-obesity, anti-thrombotic, pulmonary or cardiovascular medications (i.e. individuals to whom vaccination was routinely recommended); all other individuals were regarded as having lower-risk. We estimated incidence rate ratios with 95% CI using conditional Poisson regression in the pre-defined risk periods up to 180 days following vaccination compared to an unexposed time-period, with adjustment for season or daily temperature.

**Results:**

Overall, we observed lower risk for cardiovascular events following influenza vaccination. When stratified by baseline risk, we observed lower risk across all three outcomes in association with vaccination among higher-risk individuals. In this subgroup, relative risks were 0.72 (0.59–0.88) for AMI, 0.77 (0.59–0.99) for stroke, and 0.73 (0.45–1.19) for pulmonary embolism in the period 1–14 days following vaccination when compared to the background period. These associations remained essentially the same up to 180 days after vaccination. In contrast, the corresponding relative risks among subjects not using medications were 4.19 (2.69–6.52), 1.73 (0.91–3.31) and 2.35 (0.78–7.06).

**Conclusion:**

In this nationwide study, influenza vaccination was associated with overall cardiovascular benefit. This benefit was concentrated among those at higher cardiovascular risk as defined by medication use. In contrast, our results demonstrate no comparable inverse association with thrombosis-related cardiovascular events following vaccination among those free of cardiovascular medications at baseline. These results may inform the risk–benefit balance for universal influenza vaccination.

## Introduction

Influenza is a major trigger of cardiovascular events, and individuals with cardiovascular disease are at higher risk of influenza complications [1]. As a consequence, individuals at high cardiovascular risk, such as those with a history of cardiovascular diseases, diabetes or severe obesity, are advised to receive preventative influenza vaccination annually [2].

Although, a vaccination might trigger coagulation abnormalities [3, 4], studies showed that the benefits via preventing influenza clearly outweighs any adverse effects among individuals at high cardiovascular risk [5–11]. However, whether the benefits of the vaccine on cardiovascular events extend across the spectrum of cardiovascular risk are largely unknown.

The current US and European guidelines diverge on whether those who are apparently not at high risk should or should not be vaccinated. The Centers for Disease Control and Prevention in the US recommends that generally everyone six months of age and older should receive a vaccine [12]. In contrast, European [13] and other international guidelines [14–17] are more restrictive and advise vaccination only for those with special conditions, including high cardiovascular risk. However, none of these guidelines are based on strong evidence regarding those at lower cardiovascular risk.

In 2009, amongst fears of the pandemic influenza A(H1N1) and in contradiction to previous years, authorities recommended that the whole Norwegian population over 6 months of age undergo vaccination [18]. The vaccine was offered free of charge to all Norwegians. The high participation rate in the mass vaccination program [19] allowed us to examine the occurrence of acute myocardial infarction, stroke and pulmonary embolism following influenza vaccination among those who were apparently at lower- or higher-risk for cardiovascular events.

## Methods

### Study design

To minimize the risk of bias by unmeasured confounding, we used the self-controlled case series (SCCS) method. The SCCS method compares incidence of events across different risk periods following exposure with incidence during a baseline period. In SCCS design, cases act as their own controls in the baseline period when they are not exposed to vaccination. The SCCS method has the advantage of implicitly adjusting for all measured and unmeasured time invariant confounders, and it is considered as a standard method to study acute or triggering effects of transient exposures like vaccinations [20, 21].

### Study population

We used the Norwegian Patient Registry [22] to identify patients aged 18 years or over hospitalized for AMI, stroke or pulmonary embolism. AMI cases were identified using ICD-10 codes I21 (“acute myocardial infarction”) or I22 (“subsequent myocardial infarction”). Stroke cases were identified using ICD codes I61 (“intracerebral haemorrhage”), I63 (“cerebral infarction”) or I64 (“stroke, not specified as haemorrhage or infarction”). pulmonary embolism was defined by ICD code I26 (“pulmonary embolism”). To increase the specificity of the diagnoses, only primary diagnoses were included, and for stroke, we only included those cases with an emergency hospitalization, as previously recommended [23]. For all outcomes, we used the day of hospitalization as the date of event, and only the first episode of each event during the study period, i.e., from May 1, 2009 through September 30, 2010 was considered in our analysis. We did not include any repeated events as cardiovascular events tend to cluster within the same individual.

### Exposure information (influenza pandemic vaccine)

The exposure of interest was ‘Pandemrix’, a monovalent pandemic strain vaccine containing the oil-in-water adjuvant AS03, manufactured by GlaxoSmithKline. Information on date of vaccination was extracted from National Immunisation Registry and linked to the same individual’s previous and subsequent patient records using the Norwegian personal identification number. Generally, a single dose of Pandemrix was recommended. However, for those who received two doses, exposure was defined as the date of the first dose. The peak pandemic wave in Norway occurred between October 1, 2009 and December 31, 2009 [24]. The vaccination campaign began on October 19, 2009, and about 95% of the pandemic vaccinations were administered before December 31, 2009. The vaccination period overlapped with the peak period of the pandemic wave, but a small number of vaccines were also distributed after the peak pandemic wave. [24, 25]

### Identification of individuals at higher or lower risk for cardiovascular events

Because we did not have information on actual risk factor levels, we used medication use as a proxy for cardiovascular risk status. Information on drugs dispensed before the date of vaccination according to the Norwegian Prescription Database was used to define patients at higher- or lower-cardiovascular risk. All Norwegian pharmacies report every prescribed drug dispensed since January 1, 2004 together with the patients’ Norwegian personal identification number to this database. Patients who were dispensed any of the following drugs: anti-diabetic drugs (ATC code: A10), anti-obesity drugs (ATC code: A08), anti-thrombotic drugs (ATC code: B01), glycosides (ATC code: C01A), anti-arrhythmic drugs (ATC code: C01B), nitrates (ATC code: C01D), beta-blockers (ATC code: C07), calcium channel blockers (ATC code: C08), anti-hypertensive drugs (ATC code: C02), diuretics (ATC code: C03), peripheral vasodilators or other vasodilators (ATC code: C04), drugs acting on the renin-angiotensin system, vasoprotective drugs (ATC code: C05), lipid modifying agents (ATC code: C10) and drugs for obstructive airway diseases (ATC code: R03) were regarded as being at higher risk for cardiovascular events. In the absence of prescriptions for these drugs, we regarded participants as being at lower cardiovascular risk.

### Statistical analysis

**Fig. 1:**
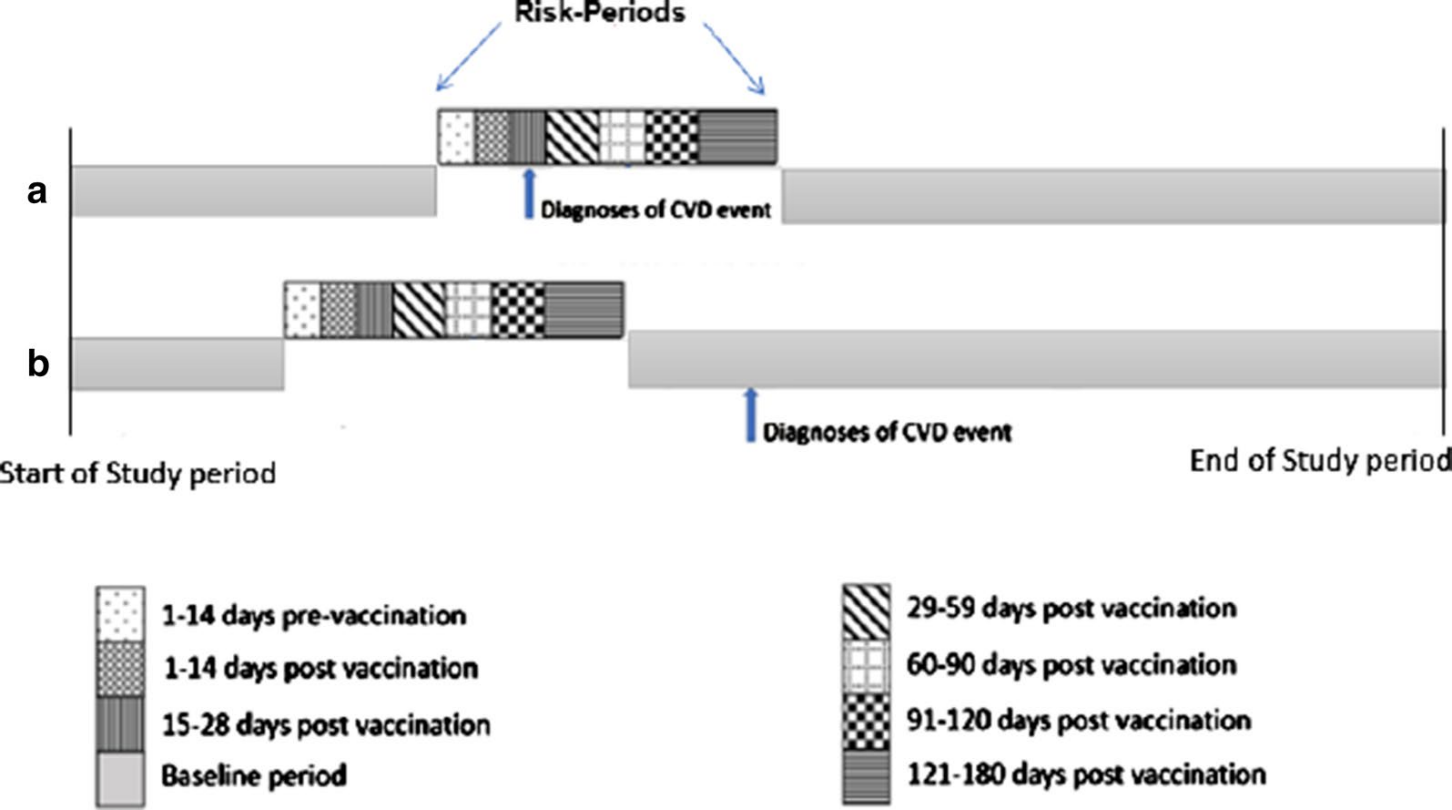
Pictorial presentation of the self-controlled case-series method applied in our study. Two possible scenarios for the timing of cardiovascular events (AMI or Stroke or Pulmonary embolism) and vaccination are shown. **a** Participant is followed from the start of the study period, has a CVD event at risk-period following vaccination. **b** Participant is followed from the start of the study period, has a CVD event at baseline following vaccination

For each individual, the observation period started from May 1, 2009 through September 30, 2010, or date of emigration or death (which ever came first). Only individuals who experienced a cardiovascular event and were vaccinated during the study period were included. Only individuals who experienced a cardiovascular event and were vaccinated during the study period were included. Exposed person time was categorised into the following risk windows: (1) from 1 to 14 days before being vaccinated (pre-vaccination ‘risk’ period), (2) from 1 to 14 days after vaccination, (3) from 15 to 28 days after vaccination, (4) from 29 to 59 days after vaccination, (5) from 60 to 90 days after vaccination, (6) from 91 to 120 days after vaccination and (7) from 120 to 180 days after vaccination. All remaining time within the observation period was used as background period (unexposed person-time), to which the exposed risk-windows were compared. The unexposed person-time may have occurred before or after vaccination within the observation period. Each vaccinated case contributed to both exposed and unexposed person-times, and the incidence-rate ratio was calculated by comparing the rate of AMI, stroke and pulmonary embolism experienced during risk periods (exposed person-time) following pandemic vaccination with the rate of these events during baseline periods (unexposed person-time) using conditional Poisson regression models. A pictorial presentation of SCCS study observation period is present in Fig. 1.

We excluded cases where the date of vaccination and date of event was on the same day as it was not clear which happened first. We present the number of these cases in Tables 2, 3 and Additional file 1: etable 1–6. As both cardiovascular events and influenza cases show seasonal variation, we adjusted for calendar period by dividing the risk periods into three calendar seasons (January-March; April-August and September-December) which reflect Norwegian seasons. All analyses were conducted separately and together for both higher- or lower-risk group defined based on medication use.

We also performed sensitivity analyses by controlling for daily mean temperature as a time-variant confounder. We extracted daily mean temperature from north, east, west, south and central regions of Norway from the webpage of the Norwegian Meteorology Institute and adjusted for temperature instead of calendar season in these additional analyses. In addition, we also performed sensitivity analyses including (1) both higher- and lower-risk group together, (2) 1–59 days as the pre-vaccination interval, (3) 15–28 days as the pre-vaccination interval, (4) 91–120 days and 121–180 days post vaccination period as part of the baseline period, and (5) both vaccinated and unvaccinated individuals during the defined study period.

In a separate analysis, we performed subgroup analysis by age (≥ 65 years versus < 65 years) for those at higher and lower cardiovascular risk. Finally, we performed stratified analyses among individuals defined as at higher risk by use of anti-platelets and anticoagulants (to determine if they would further modify risk).

Analyses were performed using Stata 16 software (StataCorp. 2016. Stata Statistical Software: Release 16. College Station, TX: StataCorp LP).

## Results

We documented 5524 vaccinated individuals who suffered an AMI, 3434 with a stroke, and 994 with pulmonary embolism during the study period who were included in our analyses. Among them 495, 308, and 136 individuals, respectively, were regarded as being at lower risk for cardiovascular events on the basis of their medication use history. Table 1 presents the general characteristics of cases of AMI, stroke and pulmonary embolism identified during the study period. When we pooled the higher- and lower-risk groups together, we observed overall reduced relative risks for AMI, stroke and pulmonary embolism following vaccination (Additional file 1: etable 1).

**Table 1:** Characteristics of the Study population

	AMI^a^	Stroke^a^	Pulmonary Embolism^a^
Total	5524	3434	994
Males, N (%)	3774 (68.3%)	1990 (57.9%)	510 (51.3%)
Higher-risk^b^, N (%)	5029 (91.0%)	3126 (91.1%)	859 (86.3%)
Lower-risk^c^, N (%)	495 (9.0%)	308 (8.9%)	136 (13.7%)
Mean age, years	67.2 (12.4)	69.9 (12.5)	63.4 (16.8)

*AMI* acute myocardial infarction, *SD* standard deviation

^a^Only first-time AMI or Stroke or Pulmonary embolism cases occurring from May 1st, 2009 through 30th September 2010 or the day of emigration or death (whichever came first) were included

^b^Higher risk was defined as being registered in the Norwegian Prescription Database for any of the following drugs: anti-diabetic drugs, anti-obesity drugs, anti-thrombotic drugs, glycosides, anti-arrhythmic drugs, nitrates, beta-blockers, calcium channel blockers, anti-hypertensive drugs, diuretics, peripheral vasodilators or other vasodilators, drugs acting on the renin-angiotensin system, vasoprotective drugs, lipid modifying agents and drugs for obstructive airway diseases from 1st January 2004 until vaccination

^c^In absence of prescriptions to these drugs, we defined individuals as being at lower risk

When we examined those aged 65 years and above and those below this age separately, the relative risks for AMI and stroke tended somewhat lower among those below 65 years than among those at 65 or above for the high-risk group (Additional file 1: etable 2). The results were generally similar for the two age groups among lower-risk individuals (Additional file 1: etable 3). When we performed analyses including 15–28 days or 1–59 days pre-vaccination interval(s), the relative risks during this interval(s) for AMI, stroke and pulmonary embolism were relatively higher (Additional file 1: etable 4 and Additional file 1: etable 5) compared to the 1–14 days pre-vaccination interval in the main analysis. Moreover, the results were similar to our main analyses when we considered 91–180 days post-vaccination as part of the baseline interval (Additional file 1: etable 5) and including both vaccinated and unvaccinated individuals (results not shown).

Given that vaccination was associated with lower relative cardiovascular risk among individuals with higher baseline risk (Table 2), but the opposite among individuals with lower baseline risk (Table 3), we examined whether antithrombotic therapy (which was, by definition, restricted to those at higher risk) might modify the acute, potentially thrombosis-related risk associated with vaccination. Consistent with this hypothesis, users of anti-platelets had considerably lower relative risks for AMI and stroke, and users of anticoagulants had considerably lower risks for pulmonary embolism than did non-users (Tables 4, 5, respectively). When we performed the analyses stratifying anti-platelet users or anticoagulants users versus non-users (both antiplatelets and anticoagulants), the results did not change (see Additional file 1: etable 6). When, instead of the season, we adjusted for daily mean temperature, the results were largely similar to the main analyses (results not shown).

**Table 2:** Incident rate ratios^a^, with 95% confidence intervals (CIs) for cardiovascular events^a^ following pandemic influenza vaccination (pandemrix) among higher risk individuals defined on the basis of medication use

	AMI (n = 5032)			Stroke (n = 3129)			Pulmonary Embolism (n = 859)		
	No. of events	Person-days at risk	IRR (95% CI)^b^	No. of events	Person-days at risk	IRR (95% CI)^b^	No. of events	Person-days at risk	IRR (95% CI)^b^
Baseline period^a^	3368	1,635,876	1.00 (ref.)	2061	1,017,036	1.00 (ref.)	564	279,612	1.00 (ref.)
Pre-vaccination interval									
1–14 days	124	70,686	0.85 (0.70–1.02)	55	43,946	0.66 (0.50–0.87)	30	12,082	1.23 (0.83–1.81)
On the day of vaccination	3	5049	0.29 (0.09–0.89)	3	3139	0.50 (0.16–1.56)	0	863	No estimates
Post vaccination interval									
1–14 days	105	70,686	0.72 (0.59–0.88)	64	43,946	0.77 (0.59–0.99)	18	12,082	0.73 (0.45–1.19)
15–28 days	101	70,686	0.70 (0.57–0.85)	80	43,946	0.96 (0.76–1.22)	22	12,082	0.89 (0.57–1.38)
29–59 days	277	156,519	0.88 (0.77–1.00)	181	97,309	0.99 (0.84–1.18)	49	26,753	0.86 (0.62–1.20)
60–90 days	261	156,519	0.87 (0.73–1.04)	171	97,309	0.96 (0.77–1.20)	39	26,753	0.65 (0.42–1.00)
91–120 days	293	141,470	1.01 (0.85–1.20)	167	94,170	0.96 (0.78–1.19)	49	25,890	0.84 (0.56–1.26)
121–180 days	500	302,940	0.82 (0.74–0.91)	347	188,340	0.92 (0.82–1.04)	88	51,780	0.81 (0.63–1.04)

*AMI* acute myocardial infarction, *IRR* incident rate ratio, *CI* confidence interval

^a^These are results from self-controlled case series analysis using data for first-time AMI (n = 5032), stroke (n = 3129) and pulmonary embolism (n = 859) patients who were vaccinated with Pandemrix. The start of observation period was May 1, 2009 and end of observation period was September 30, 2010 or the day of emigration or death (whichever came first). Person-time of each vaccinated individual was divided into following risk-periods: pre-vaccination interval (1–14 days prior to vaccination) and postvaccination intervals (1–14 days, 15–28 days, 29–59 days, 60–90 days, 91–120 days, 121–180 days following vaccination)

^b^All remaining part of the observation period was used for baseline comparison (unexposed person-time)

^c^Adjusted for calendar period (January–March, April–August, and September–December)

**Table 3:** Incident rate ratios, with 95% confidence intervals (CIs) for cardiovascular events following pandemic influenza vaccination (pandemrix) among lower risk individuals

	AMI (n = 495)			Stroke (n = 308)			Pulmonary embolism (n = 136)		
	No. of events	Person-days at risk	IRR (95% CI)^b^	No. of events	Person-days at risk	IRR (95% CI)^b^	No. of events	Person-days at risk	IRR (95% CI)^b^
Baseline period^a^	209	160,056	1.00 (ref.)	160	99,468	1.00 (ref.)	59	44,064	1.00 (ref)
Pre-vaccination interval									
1–14 days	2	6916	0.29 (0.07–1.19)	4	4298	0.63 (0.23–1.74)	1	1904	0.59 (0.08–4.50)
On the day of vaccination	–	–	No estimates	–	–	No estimates	–	–	No estimates
Post-vaccination interval									
1–14 days	29	6916	4.19 (2.69–6.52)	11	4298	1.73 (0.90–3.31)	4	1904	2.35 (0.78–7.06)
15–28 days	24	6916	3.17 (1.99–5.07)	10	4298	1.51 (0.77–2.97)	5	1904	2.61 (0.96–7.08)
29–59 days	43	15,314	2.28 (1.50–3.49)	22	9517	1.40 (0.81–2.42)	7	4216	1.40 (0.56–3.52)
60–90 days	57	15,314	2.78 (1.79–4.32)	31	9517	1.86 (1.04–3.32)	20	4216	3.43 (1.57–7.47)
91–120 days	46	14,820	2.27 (1.50–3.44)	22	9210	1.37 (0.76–2.46)	18	4080	3.10 (1.50–6.41)
121–180 days	85	29,640	1.99 (1.54–2.59)	48	18,420	1.54 (1.10–2.17)	22	8160	1.80 (1.08–3.00)

These are results from self-controlled case series analysis using data for first-time AMI (n = 495), stroke (n = 308) and pulmonary embolism (n = 136) patients who were vaccinated with Pandemrix. The start of observation period was May 1, 2009 and end of observation period was September 30, 2010 or the day of emigration or death (whichever came first). Person-time of each vaccinated individual was divided into following risk-periods: pre-vaccination interval (1–14 days prior to vaccination) and postvaccination intervals (1–14 days, 15–28 days, 29–59 days, 60–90 days, 91–120 days, 121–180 days following vaccination)

*AMI* acute myocardial infarction, *IRR* incident rate ratio, *CI* confidence interva

^a^All remaining part of the observation period was used for baseline comparison (unexposed person-time)

^b^Adjusted for calendar period (January-March, April-August, and September-December)

**Table 4:** Incident rate ratios, with 95% confidence intervals (CIs) for cardiovascular events following pandemic influenza vaccination (pandemrix) among higher risk individuals, stratified by antiplatelet users versus non-users

		AMI^a^				Stroke^a^				Pulmonary embolism^a^		
		Anti-platelet users (n = 3932)		Antiplatelet non-users (n = 1097)		Anti-platelet users (n = 2203)		Antiplatelet non users (n = 923)		Anti-platelet users (n = 319)		Antiplatelet non users (n = 540)
	N	IRR (95% CI)^c^	N	IRR (95% CI)^c^	N	IRR (95% CI)^c^	N	IRR (95% CI)^c^	N	IRR (95% CI)^c^	N	IRR (95% CI)^c^
Baseline period^b^	2921	1.0 (ref.)	443	1.0 (ref.)	1561	1.00 (ref.)	500	1.0 (ref.)	194	1.00 (ref.)	370	1.0 (ref)
Pre vaccination interval												
1–14 days	117	0.87 (0.71–1.06)	8	0.51 (0.25–1.05)	45	0.69 (0.51–0.94)	10	0.51 (0.27–0.98)	7	0.92 (0.42–2.01)	23	1.37 (0.88–2.14)
Post vaccination interval												
1–14 days	55	0.41 (0.31–0.54)	50	3.28 (2.37–4.54)	39	0.60 (0.43–0.84)	25	1.29 (0.84–1.97)	10	1.30 (0.66–2.56)	8	0.48 (0.23–0.98)
15–28 days	64	0.48 (0.37–0.62)	37	2.35 (1.64–3.38)	45	0.70 (0.52–0.96)	35	1.78 (1.24–2.57)	6	0.76 (0.33–1.77)	16	0.96 (0.57–1.61)
29–59 days	167	0.58 (0.49–0.68)	111	3.15 (2.46–4.03)	114	0.84 (0.68–1.03)	67	1.50 (1.12–2.01)	23	1.19 (0.72–1.98)	26	0.71 (0.45–1.10)
60–90 days	160	0.59 (0.47–0.75)	101	2.77 (2.08–3.70)	94	0.74 (0.56–0.99)	76	1.62 (1.15–2.29)	20	0.86 (0.45–1.64)	19	0.52 (0.29–0.95)
91–120 days	169	0.65 (0.52–0.82)	124	3.34 (2.55–4.36)	98	0.79 (0.60–1.05)	70	1.53 (1.09–2.16)	25	1.10 (0.59–2.04)	24	0.69 (0.40–1.19)
121–180 days	279	0.53 (0.46–0.61)	220	2.64 (2.22–3.13)	207	0.74 (0.64–0.87)	140	1.48 (1.21–1.81)	34	0.85 (0.56–1.28)	54	0.79 (0.58–1.09)

*AMI* acute myocardial infarction, *IRR* incident rate ratio, *CI* confidence interval

^a^Analyses was restricted among higher cardiovascular-risk patients. These are results from self-controlled case series analysis using data for first-time AMI, stroke and pulmonary embolism patients who were vaccinated with Pandemrix. The start of observation period was May 1, 2009 and end of observation period was September 30, 2010 or the day of emigration or death (whichever came first). Person-time of each vaccinated individual was divided into following risk-periods: pre-vaccination interval (1–14 days prior to vaccination) and postvaccination intervals (1–14 days, 15–28 days, 29–59 days, 60–90 days, 91–120 days, 121–180 days following vaccination)

^b^All remaining part of the observation period was used for baseline comparison (unexposed person-time)

^c^Adjusted for calendar period (January–March, April–August, and September–December)

**Table 5:** Incident rate ratios, with 95% confidence intervals (CIs) cardiovascular events following pandemic influenza vaccination (pandemrix) stratified by anticoagulants among higher risk individuals, stratified by anti-coagulants users versus non-users

		AMI^a^				Stroke^a^				Pulmonary embolism^a^		
		Anti-coagulants users (n = 892)		Anti-coagulants non-users (n = 4134)		Anti-coagulants users (n = 839)		Anti-coagulants non-users (n = 2287)		Anti-coagulants users (n = 530)		Anti-coagulants non-users (n = 329)
	N	IRR (95%CI)^c^	N	IRR (95%CI)^c^	N	IRR (95%CI)^c^	N	IRR (95%CI)^c^	N	IRR (95%CI)^c^	N	IRR (95%CI)^c^
Baseline period^b^	595	1.0 (ref.)	2767	1.00 (ref.)	550	1.00 (ref.)	1517	1.0 (ref.)	425	1.00 (ref.)	139	1.0 (ref.)
Pre vaccination interval												
1–14 days	20	0.78 (0.49–1.25)	103	0.85 (0.69–1.04)	18	0.82 (0.50–1.33)	37	0.60 (0.43–0.83)	28	1.41 (0.94–2.11)	2	0.40 (0.10–1.67)
Post vaccination interval												
1–14 days	15	0.59 (0.35–1.00)	90	0.74 (0.60–0.92)	15	0.68 (0.40–1.16)	49	0.79 (0.59–1.06)	5	0.25 (0.10–0.61)	13	2.61 (1.40–4.89)
15–28 days	14	0.55 (0.32–0.95)	88	0.73 (0.59–0.91)	25	1.14 (0.75–1.74)	55	0.89 (0.67–1.18)	6	0.30 (0.13–0.68)	16	3.12 (1.76–5.53)
29–59 days	50	0.92 (0.67–1.27)	229	0.88 (0.75–1.02)	47	0.99 (0.71–1.39)	134	0.99 (0.81–1.21)	11	0.25 (0.13–0.47)	38	3.09 (1.99–4.78)
60–90 days	60	1.20 (0.80–1.79)	203	0.81 (0.67–0.99)	50	1.12 (0.74–1.70)	119	0.89 (0.69–1.16)	8	0.17 (0.07–0.42)	31	2.21 (1.30–3.75)
91–120 days	50	1.03 (0.68–1.57)	244	1.01 (0.84–1.22)	40	0.92 (0.59–1.43)	127	0.98 (0.76–1.25)	21	0.48 (0.24–0.95)	28	2.04 (1.20–3.46)
121–180 days	88	0.83 (0.65–1.07)	410	0.82 (0.73–0.92)	96	0.97 (0.77–1.23)	249	0.90 (0.78–1.03)	26	0.32 (0.21–0.51)	62	2.25 (1.63–3.10)

*AMI* acute myocardial infarction, *IRR* incident rate ratio, *CI* confidence interval

^a^Analyses was restricted among higher cardiovascular-risk patients. These are results from self-controlled case series analysis using data for first-time AMI, stroke and pulmonary embolism patients who were vaccinated with Pandemrix. The start of observation period was May 1, 2009 and end of observation period was September 30, 2010 or the day of emigration or death (whichever came first). Person-time of each vaccinated individual was divided into following risk-periods: pre-vaccination interval (1–14 days prior to vaccination) and postvaccination intervals (1–14 days, 15–28 days, 29–59 days, 60–90 days, 91–120 days, 121–180 days following vaccination)

^b^All remaining part of the observation period was used for baseline comparison (unexposed person-time)

^c^Adjusted for calendar period (January–March, April–August, and September–December)

## Discussion

In this nationwide study, which included all Norwegian residents who sustained an AMI, stroke or pulmonary embolism within 180 days of a broadly recommended influenza vaccination, we observed a lower risk for all three cardiovascular events following vaccination among those at higher cardiovascular risk. This finding generally concords with previous studies, [5–7, 9–11, 26, 27] which included exclusively or predominantly high-risk individuals. In our study, those taking antithrombotic medication appeared to benefit most clearly from vaccination. In contrast, among those having no prescribed cardiovascular medication at baseline, vaccination appeared to be associated with acutely increased cardiovascular risk.

Few population-based studies have been able to assess the association between influenza vaccine and risk for AMI, stroke and pulmonary embolism among those at lower cardiovascular risk. This study was uniquely possible due to the special situation that occurred during the influenza A (H1N1) pandemic [19]. Contrary to preceding and subsequent years, in 2009, the entire Norwegian population was advised to receive the vaccine, and vaccinations were easily available through a public vaccination campaign. As a result, 43% of the population was vaccinated [19]. In contrast, only around 10% of the population is vaccinated against influenza during ordinary seasons. [28]

Current guidelines across the globe generally emphasize the importance of influenza vaccination among high-risk groups. [13–16] Before 2010, the Centers for Disease Control and Prevention in the US had a similar recommendation. However, a meeting of the Advisory Committee on Immunization Practices changed this approach due to the fears of circulating H1N1 pandemic virus and because many were likely to be unaware of their high risk status [29]. Accordingly, the current recommendation in the US is to vaccinate all individuals aged 6 months and older [12].

In this study, we examined the risk for AMI, stroke and pulmonary embolism after an influenza vaccination among those at no apparent increased risk for cardiovascular events based upon comprehensive drug registers. Our findings do not necessarily support the extension of vaccination to lower-risk individuals, at least for cardiovascular prevention, as we found no cardiovascular benefit of the vaccine among those not taking no prescribed cardiovascular medications. On the contrary, we unexpectedly found an increased risk for cardiovascular events after vaccination compared to the baseline period among these people. At the same time, it is likely that other benefits may exist from vaccination of lower-risk individuals, including fewer days of work lost due to illness and greater spread of herd immunity.

Our results concerning those whom we categorized as having low cardiovascular risk may reflect study design issues. As the SCCS is restricted to those with an event, and we only considered one event per individual, excluding those with medication use at the time of vaccination increases the likelihood that the event happens after vaccination as opposed to before, which in turn increases the relative risk. Moreover, the self-controlled case series method is susceptible to confounding by factors that vary within the individual. With a vaccination rate below 50%, vaccination was still somewhat selective, rather than truly universal, during this time period among lower-risk individuals. It is possible that a small number of these individuals were vaccinated specifically because their health was declining, leading to an artificially increased rate of events in the subsequent months. For this to be true, no similar decline could have been present among higher-risk individuals. Additional studies with access to detailed medical record data will be needed to evaluate this possibility.

On the other hand, if our findings reflect a true causal effect of the vaccine, the potential mechanism could be an alteration in the immune system following adjuvant-boosted vaccination that might trigger hypercoagulability—a common denominator for AMI, ischemic stroke and pulmonary embolism [30]. However, previous studies suggest that any such effect is likely be transient [31], while we observed an elevated risk even several months later. We can only speculate that, at least in a subset of susceptible individuals, vaccination might induce a more sustained effect. Of note, when we stratified higher-risk individuals according to the use of anti-thrombotic medication, those on anti-platelets were at considerably lower risk for AMI and stroke and those on anti-coagulants were at a considerably lower risk for pulmonary embolism than those not using these medications, respectively. These findings are consistent with the hypothesis that anti-platelets and anti-coagulants prevent coagulation in the high- and low-pressure parts of the circulatory system, respectively [32], although neither treatment can be recommended specifically to prevent vaccination-related complications without further clinical study.

Our study had several important limitations. Our most important limitation is that, contrary to some previous similar studies [5, 6, 10], we included only a single season in our analyses and variations in incidence of cardiovascular events occurring during the study period could have affected our estimates. However, it is unlikely that such variations affected lower- and higher-risk individuals differentially, and thus is unlikely to explain the clear contrast in observed risk after vaccination in these strata.

Stable characteristics like age, sex, education or chronic comorbidities that might affect both participation in the vaccination program and risk for cardiovascular events are not likely to explain the observed associations. We used SCCS analyses, and we performed within-person comparisons that implicitly control for unmeasured time-invariant confounders. On the other hand, this design is not immune to the so-called immortal time bias, which may arise when a prerequisite to inclusion to the study is to survive until vaccination. This typically causes an upwards bias of estimates [33]. However, it is not clear how this bias, could explain the differential associations for lower- and higher-risk groups. This method only produces estimates of risk relative to an individual’s baseline, which is likely to be quite modest among lower-risk individuals.

One of the assumptions of the SCCS is that the event of interest does not increase the probability of death. Cardiovascular events are known to increase mortality, and thus this assumption was violated in our study. However, this could not possibly explain the marked difference in the observed effect of the vaccination among high- and low-risk individuals. Previous studies using SCCS to study the effects of influenza vaccination on risk for CVD events examined the size of the bias due this violation of assumptions and concluded that it is likely to be negligible [10, 34].

As cardiovascular events tend to cluster within the same individual, similar to previous studies, we disregarded repeated events during the follow up. However, this approach might have led to some, most probably minor, distortion of our results.

We utilized data from national registers. Registration of vaccination, dispensing of prescribed medications, and hospitalizations were mandatory, which ensured independent reporting and an unbiased assessment of the outcomes during follow up. The Norwegian Patient Registry has virtually complete coverage of medical and surgical care at hospitals and a generally high accuracy of diagnoses [23, 35]. To increase specificity, we restricted to cases with a primary diagnosis. In the Norwegian Patient Registry, the primary diagnosis is given to the main reason for the patient’s need for treatment or investigation at a given hospitalization, and it has higher specificity than secondary diagnoses. On the other hand, our sensitivity was apt to be imperfect as we missed cases who died before arrival or, due to other reasons, were not treated at hospitals. However, specificity is generally a greater concern than sensitivity in similar settings [36].

Our definition of higher- and lower-cardiovascular risk had some limitations. We relied only on information on prescribed medications for conditions known to elevate cardiovascular risk. Therefore, the results for the lower-risk individuals should be interpreted with *caution* in the absence of information on actual risk factor levels. Many may not receive any pharmaceutical treatment for such conditions, although, it should be emphasized that, in Norway, health care is equally available to all citizens irrespective of their income. Moreover, the Norwegian Prescription Database contains information only from pharmacies, and has no information on drugs given to institutionalized patients such as those in nursing homes.

Finally, it is not clear to what extent our results on use of Pandemrix during the H1N1 pandemic in 2009–2010 generalizes to other seasons with different vaccines and different influenza strains.

## Conclusion

This study confirms previous studies suggesting that patients at higher cardiovascular risk appear to benefit from pandemic influenza vaccination. The protective effect of the vaccination was especially pronounced among those taking antithrombotic medication. However, our findings provide no support for a general recommendation of vaccination to everyone for prevention of cardiovascular outcomes. On the contrary, pandemic vaccination appeared to increase the acute risk of AMI, stroke and pulmonary embolism among those who were initially free of cardiovascular drugs. Further studies that are able to define lower- and higher-risk groups based on pre-existing diagnoses, rather than medications, would be of great relevance.

## Supplementary Information

**Additional file 1**. eTable1 to eTable6.

## Data Availability

The registry datasets that support the findings of this study are available from the Health authorities in Norway, but restrictions apply regarding the availability of these data, which were originally used under specific approvals for the current study and are therefore not publicly available. Data are, however, available after approval by data owners and ethical committees (https://helsedata.no/en/personally-identifiable-data/) and ethical committees (https://rekportalen.no/#hjem/home). Statistical codes used for generating results can be made available from the corresponding author AS on reasonable request.

## Abbreviations

AMI: Acute myocardial infarction
ATC: Anatomical Therapeutic Chemical Classification
PE: Pulmonary embolism
SCCS: Self-controlled case-series
