# Supplementary material for: Influenza vaccination and risk for cardiovascular events: a nationwide self-controlled case series study
Source: BMC Cardiovasc Disord. 2021 Jan 13;21:31. doi: 10.1186/s12872-020-01836-z (PMC7803467; doi:10.1186/s12872-020-01836-z)
Supplement: Supplementary file 1 — Additional file 1. eTable1 to eTable6. [file 12872_2020_1836_MOESM1_ESM.docx]

**Supplementary Table(s)**

| eTable 1 Incident rate ratios , with 95% confidence intervals (CIs) for cardiovascular events^a^ following pandemic influenza vaccination (pandemrix) | | | | | | | | | | | |
| --- | --- | --- | --- | --- | --- | --- | --- | --- | --- | --- | --- |
|  |  | **AMI**  **(n=5524)** |  |  | | **Stroke**  **(n=3434)** | |  |  | **Pulmonary Embolism**  **(n= 994)** |  |
|  | **No. of**  **events** | **Person-days at risk** | **IRR (95%CI)**^c^ | **No. of**  **events** | **Person-days at risk** | | **IRR (95%CI)**^c^ | | **No. of events** | **Person-**  **days**  **at risk** | **IRR (95%CI)**^c^ |
| Baseline period^b^ | 3578 | 1793988 | 1.00 | 2225 | 1116504 | | 1.00 | | 623 | 323676 | 1.00 |
| *Pre-vaccination interval* |  |  |  |  |  | |  | |  |  |  |
| 1-14 days | 125 | 77518 | 0.83 (0.69-1.00) | 59 | 48244 | | 0.66 (0.50-0.86) | | 30 | 13986 | 1.20 (0.82-1.76) |
| On the day of vaccination | 3 | - | 0.28 (0.09-0.87) | 3 | - | | 0.47 (0.15-1.46) | | - | - | *-* |
| *Post vaccination interval* |  |  |  |  |  | |  | |  |  |  |
| 1-14 days | 133 | 77518 | 0.88 (0.74-1.06) | 75 | 48244 | | 0.84 (0.66-1.06) | | 22 | 13986 | 0.85 (0.55-1.33) |
| 15-28 days | 125 | 77518 | 0.83 (0.69-1.00) | 90 | 48244 | | 1.01 (0.81-1.26) | | 27 | 13986 | 1.03 (0.69-1.54) |
| 29-59 days | 322 | 171647 | 0.97 (0.85-1.10) | 202 | 106826 | | 1.03 (0.87-1.21) | | 56 | 30969 | 0.93 (0.68-1.27) |
| 60-90 days | 314 | 171647 | 0.96 (0.81-1.13) | 200 | 106826 | | 1.03 (0.84-1.27) | | 59 | 30969 | 0.92 (0.64-1.34) |
| 91-120 days | 337 | 166110 | 1.06 (0.90-1.24) | 190 | 103380 | | 1.00 (0.82-1.23) | | 67 | 29970 | 1.08 (0.76-1.54) |
| 121-180 days | 588 | 332220 | 0.90 (0.81-0.99) | 390 | 206760 | | 0.96 (0.86-1.08) | | 110 | 59940 | 0.92 (0.74-1.15) |
| AMI: acute myocardial infarction IRR: incident rate ratio CI: confidence interval  ^a^These are results from self-controlled case series analysis using data for first-time AMI (n=5032), stroke (n=3129) and pulmonary embolism (n=859) patients who were vaccinated with Pandemrix. The start of observation period was May 1, 2009 and end of observation period was September 30, 2010 or the day of emigration or death (whichever came first). Person-time of each vaccinated individual was divided into following risk-periods: pre-vaccination interval (1-14 days prior to vaccination) and postvaccination intervals (1-14 days, 15-28 days, 29-59 days, 60-90 days, 91-120 days, 121-180 days following vaccination).  ^b^ All remaining part of the observation period was used for baseline comparison (unexposed person-time).  ^c^adjusted for calendar period (January-March, April-August, and September-December) | | | | | | | | | | | |

| eTable 2. Incident rate ratios , with 95% confidence intervals (CIs) for different cardiovascular events^a^ following pandemic influenza vaccination (pandemrix) among higher cardiovascular risk patients, a subgroup analysis by age (<65years and ≥65 years)^a^ | | | | | | | | | | | | |
| --- | --- | --- | --- | --- | --- | --- | --- | --- | --- | --- | --- | --- |
|  | **AMI** | | | | **Stroke** | | | | **Pulmonary Embolism** | | | |
|  | **Age<65 years**  **(n=2163)** | | **Age≥65 years**  **(n=2866)** | | **Age<65 years**  **(n=893)** | | **Age≥65 years**  **(n=2233)** | | **Age<65 years**  **(n=377)** | | **Age≥65 years**  **(n=482)** | |
|  | **No. of Events** | **IRR (95% CI)^c^** | **No. of Events** | **IRR (95% CI)^c^** | **No. of Events** | **IRR (95% CI)^c^** | **No. of Events** | **IRR (95% CI)^c^** | **No. of Events** | **IRR (95% CI)^c^** | **No. of Events** | **IRR (95% CI)^c^** |
| Baseline period^b^ | 1540 | 1.00 (ref.) | 1825 | 1.00 (ref.) | 622 | 1.00 (ref.) | 1444 | 1.00 (ref) | 252 | 1.00 (ref) | 312 | 1.00 (ref) |
| *Pre vaccination interval* |  |  |  |  |  |  |  |  |  |  |  |  |
| 1-14 days | 77 | 1.06 (0.83-1.35) | 47 | 0.62 (0.46-0.84) | 15 | 0.53 (0.31-0.90) | 40 | 0.71 (0.52-0.99) | 14 | 1.21 (0.69-2.14) | 15 | 1.15 (0.67-1.99) |
| *Post vaccination interval* |  |  |  |  |  |  |  |  |  |  |  |  |
| 1-14 days | 41 | 0.57 (0.41-0.78) | 64 | 0.85 (0.66-1.11) | 21 | 0.74 (0.47-1.17) | 43 | 0.77 (0.56-1.05) | 8 | 0.69 (0.34-1.44) | 10 | 0.77 (0.40-1.47) |
| 15-28 days | 39 | 0.54 (0.39-0.76) | 63 | 0.84 (0.65-1.09) | 19 | 0.66 (0.41-1.06) | 61 | 1.10 (0.84-1.44) | 10 | 0.89 (0.46-1.71) | 12 | 0.88 (0.49-1.61) |
| 29-59 days | 97 | 0.63 (0.51-0.79) | 181 | 1.09 (0.92-1.30) | 46 | 0.69 (0.50-0.97) | 135 | 1.14 (0.93-1.39) | 19 | 0.79 (0.48-1.32) | 30 | 0.90 (0.58-1.40) |
| 60-90 days | 94 | 0.69 (0.52-0.93) | 167 | 1.01 (0.82-1.27) | 46 | 0.63 (0.41-0.95) | 124 | 1.11 (0.86-1.44) | 19 | 0.88 (0.46-1.68) | 20 | 0.51 (0.28-0.93) |
| 91-120 days | 108 | 0.83 (0.62-1.11) | 185 | 1.16 (0.93-1.43) | 39 | 0.55 (0.36-0.85) | 129 | 1.17 (0.92-1.50) | 18 | 0.86 (0.45-1.65) | 31 | 0.83 (0.49-1.39) |
| 121-180 days | 167 | 0.62 (0.52-0.74) | 335 | 1.00 (0.88-1.13) | 84 | 0.69 (0.53-0.89) | 262 | 1.01 (0.88-1.16) | 37 | 0.84 (0.57-1.22) | 51 | 0.80 (0.57-1.11) |
| AMI: acute myocardial infarction IRR: incident rate ratio CI: confidence interval  ^a^Analyses was restricted among higher cardiovascular risk patients. These are results from self-controlled case series analysis using data for first-time AMI, stroke and pulmonary embolism patients who were vaccinated with Pandemrix. The start of observation period was May 1, 2009 and end of observation period was September 30, 2010 or the day of emigration or death (whichever came first). Person-time of each vaccinated individual was divided into following risk-periods: pre-vaccination interval (1-14 days prior to vaccination) and post-vaccination intervals (1-14 days, 15-28 days, 29-59 days, 60-90 days, 91-120 days, 121-180 days following vaccination).  ^b^ All remaining part of the observation period was used for baseline comparison (unexposed person-time).  ^c^ Adjusted for calendar period (January-March, April-August, and September-December) | | | | | | | | | | | | |

| eTable 3. Incident rate ratios , with 95% confidence interval (CIs) for different cardiovascular events^a^ following pandemic influenza vaccination (pandemrix) among lower cardiovascular risk patients, a subgroup analysis by age (<65years and ≥65 years) | | | | | | | | | | | | |
| --- | --- | --- | --- | --- | --- | --- | --- | --- | --- | --- | --- | --- |
|  | **AMI** | | | | **Stroke** | | | | **Pulmonary Embolism** | | | |
|  | **Age <65 years**  **(n=305)** | | **Age≥65 years**  **(n=192)** | | **Age <65 years**  **(n=170)** | | **Age≥65 years**  **(n=138)** | | **Age<65 years**  **(n=97)** | | **Age≥65 years**  **(n=38)** | |
|  | **No. of Events** | **IRR (95% CI)^c^** | **No. of Events** | **IRR (95% CI)^c^** | **No. of Events** | **IRR (95% CI)^c^** | **No. of Events** | **IRR (95% CI)^c^** | **No. of Events** | **IRR (95% CI)^c^** | **No. of Events** | **IRR (95% CI)^c^** |
| Baseline period^b^ | 132 | 1.00 (ref.) | 77 | 1.00 (ref.) | 96 | 1.00 (ref.) | 64 | 1.00 (ref) | 43 | 1.00 (ref) | 15 | 1.00 (ref) |
| *Pre vaccination interval* |  |  |  |  |  |  |  |  |  |  |  |  |
| 1-14 days | 1 | 0.24 (0.03-1.76) | 1 | 0.34 (0.05-2.50) | 3 | 0.75 (0.23-2.46) | 1 | 0.39 (0.05-2.89) | - | - | 1 | 2.01 (0.23-17.48) |
| *Post vaccination interval* |  |  |  |  |  |  |  |  |  |  |  |  |
| 1-14 days | 18 | 4.33 (2.46-7.62) | 10 | 3.54 (1.63-7.00) | 7 | 1.76 (0.77-3.99) | 4 | 1.53 (0.53-4.43) | 3 | 2.49 (0.69-8.96) | 1 | 1.99 (0.23-16.94) |
| 15-28 days | 15 | 3.35 (1.85-6.07) | 9 | 2.90 (1.31-5.94) | 5 | 1.28 (0.50-3.27) | 5 | 1.70 (0.64-4.53) | 4 | 2.96 (0.95-9.20) | 1 | 1.78 (0.21-15.13) |
| 29-59 days | 22 | 2.00 (1.14-3.51) | 22 | 2.83 (1.45-5.21) | 10 | 1.21 (0.57-2.60) | 11 | 1.44 (0.64-3.29) | 3 | 0.85 (0.23-3.16) | 4 | 2.74 (0.68-11.12) |
| 60-90 days | 32 | 2.76 (1.57-4.87) | 25 | 2.88 (1.47-5.79) | 14 | 1.78 (0.77-4.12) | 17 | 2.02 (0.89-4.61) | 18 | 4.36 (1.75-10.88) | 2 | 1.22 (0.20-7.30) |
| 91-120 days | 26 | 2.17 (1.26-3.72) | 20 | 2.50 (1.28-4.68) | 10 | 1.29 (0.55-3.05) | 12 | 1.54 (0.68-3.48) | 10 | 2.42 (0.96-6.09) | 8 | 5.00 (1.49-16.75) |
| 121-180 days | 58 | 2.16 (1.57-2.98) | 29 | 1.90 (1.21-2.95) | 25 | 1.43 (0.90-2.28) | 24 | 1.77 (1.07-2.93) | 16 | 1.77 (0.97-3.23) | 6 | 1.92 (0.72-5.12) |
| AMI: acute myocardial infarction IRR: incident rate ratio CI: confidence interval  ^a^ Analysis was restricted to lower cardiovascular risk patients. These are results from self-controlled case series analysis using data for first-time AMI, stroke and pulmonary embolism patients who were vaccinated with Pandemrix. The start of observation period was May 1, 2009 and end of observation period was September 30, 2010 or the day of emigration or death (whichever came first). Person-time of each vaccinated individual was divided into following risk-periods: pre-vaccination interval (1-14 days prior to vaccination) and post-vaccination intervals (1-14 days, 15-28 days, 29-59 days, 60-90 days, 91-120 days, 121-180 days following vaccination).  ^b^ All remaining part of the observation period was used for baseline comparison (unexposed person-time).  ^c^ Adjusted for calendar period (January-March, April-August, and September-December) | | | | | | | | | | | | |

| eTable 4. Incident rate ratios , with 95% confidence interval (CIs) for different cardiovascular events^a^ following pandemic influenza vaccination (pandemrix) | | | | | | | | | | | | |
| --- | --- | --- | --- | --- | --- | --- | --- | --- | --- | --- | --- | --- |
|  | **AMI** | | | | **Stroke** | | | | **Pulmonary Embolism** | | | |
|  | **Higher-risk** | | **Lower-risk** | | **Higher-risk** | | **Lower-risk** | | **Higher-risk** | | **Lower-risk** | |
|  | **No. of Events** | **IRR (95% CI)^c^** | **No. of Events** | **IRR (95% CI)^c^** | **No. of Events** | **IRR (95% CI)^c^** | **No. of Events** | **IRR (95% CI)^c^** | **No. of Events** | **IRR (95% CI)^c^** | **No. of Events** | **IRR (95% CI)^c^** |
| Baseline period^b^ | 2763 | 1.00 (ref.) | 204 | 1.00 (ref.) | 1780 | 1.00 (ref.) | 146 | 1.00 (ref) | 464 | 1.00 (ref) | 55 | 1.00 (ref) |
| *Pre vaccination interval* |  |  |  |  |  |  |  |  |  |  |  |  |
| 1-59 days | 730 | 1.59 (1.41-1.79) | 5 | 0.12 (0.05-0.30) | 338 | 1.02 (0.88-1.20) | 18 | 0.58 (0.33-1.01) | 130 | 1.68 (1.27-2.22) | 4 | 0.47 (0.15-1.46) |
| On the vaccination day | 3 | 0.38 (0.12-1.19) | - | - | 3 | 0.53 (0.17-1.66) | - | - | - | - | - | - |
| *Post-vaccination interval* |  |  |  |  |  |  |  |  |  |  |  |  |
| 1-14 days | 105 | 0.95 (0.77-1.18) | 28 | 2.76 (1.75-4.36) | 64 | 0.81 (0.62-1.06) | 11 | 1.45 (0.74-2.84) | 18 | 0.96 (0.58-1.61) | 4 | 1.90 (0.61-5.91) |
| 15-28 days | 100 | 0.90 (0.73-1.12) | 24 | 2.30 (1.44-3.70) | 80 | 1.01 (0.80-1.29) | 10 | 1.29 (0.65-2.59) | 22 | 1.14 (0.71-1.82) | 5 | 2.19 (0.79-6.08) |
| 29-59 days | 278 | 1.11 (0.96-1.29) | 43 | 1.80 (1.18-2.73) | 181 | 1.04 (0.87-1.25) | 22 | 1.24 (0.71-2.16) | 49 | 1.06 (0.74-1.52) | 7 | 1.23 (0.48-3.12) |
| 60-90 days | 260 | 0.99 (0.83-1.19) | 56 | 2.28 (1.47-3.53) | 170 | 0.99 (0.79-1.23) | 31 | 1.69 (0.94-3.04) | 39 | 0.74 (0.47-1.15) | 20 | 3.13 (1.43-6.88) |
| 91-120 days | 293 | 1.14 (0.96-1.35) | 46 | 1.96 (1.29-2.98) | 168 | 1.00 (0.80-1.24) | 22 | 1.26 (0.70-2.28) | 49 | 0.94 (0.63-1.42) | 18 | 2.88 (1.38-6.01) |
| 121-180 days | 499 | 0.86 (0.78-0.95) | 87 | 1.95 (1.51-2.54) | 345 | 0.93 (0.82-1.05) | 47 | 1.48 (1.05-2.08) | 88 | 0.85 (0.66-1.09) | 22 | 1.75 (1.05-2.93) |
| AMI: acute myocardial infarction IRR: incident rate ratio CI: confidence interval  ^a^ These are results from self-controlled case series analysis using data for first-time AMI, stroke and pulmonary embolism patients who were vaccinated with Pandemrix. The start of observation period was May 1, 2009 and end of observation period was September 30, 2010 or the day of emigration or death (whichever came first). Person-time of each vaccinated individual was divided into following risk-periods: pre-vaccination interval (1-59 days prior to vaccination) and post-vaccination intervals (1-14 days, 15-28 days, 29-59 days, 60-90 days, 91-120 days, 121-180 days following vaccination). ^b^ All remaining part of the observation period was used for baseline comparison (unexposed person-time).  ^c^ Adjusted for calendar period (January-March, April-August, and September-December) | | | | | | | | | | | | |

| eTable 5. Incident rate ratios , with 95% confidence interval (CIs) for different cardiovascular events^a^ following pandemic influenza vaccination (pandemrix) | | | | | | | | | | | | |
| --- | --- | --- | --- | --- | --- | --- | --- | --- | --- | --- | --- | --- |
|  | **AMI** | | | | **Stroke** | | | | **Pulmonary Embolism** | | | |
|  | **Higher-risk** | | **Lower-risk** | | **Higher-risk** | | **Lower-risk** | | **Higher-risk** | | **Lower-risk** | |
|  | **No. of Events** | **IRR (95% CI)^c^** | **No. of Events** | **IRR (95% CI)^c^** | **No. of Events** | **IRR (95% CI)^c^** | **No. of Events** | **IRR (95% CI)^c^** | **No. of Events** | **IRR (95% CI)^c^** | **No. of Events** | **IRR (95% CI)^c^** |
| Baseline period^b^ | 3970 | 1.00 (ref.) | 342 | 1.00 (ref.) | 2495 | 1.00 (ref.) | 224 | 1.00 (ref.) | 672 | 1.00 (ref.) | 98 | 1.00 (ref) |
| *Pre vaccination interval* |  |  |  |  |  |  |  |  |  |  |  |  |
| 15-28 days | 189 | 1.49 (1.27-1.74) | - | - | 82 | 1.07 (0.85-1.35) | 5 | 0.78 (0.31-1.95) | 29 | 1.36 (0.91-2.02) | - | *-* |
| 1-14 days | 124 | 0.91 (0.75-1.10) | 2 | 0.22 (0.05-0.89) | 55 | 0.67 (0.50-0.88) | 4 | 0.57 (0.21-1.59) | 30 | 1.20 (0.81-1.78) | 1 | 0.59 (0.08-4.50) |
| On the vaccination day | 3 | 0.31 (0.10-0.96) | - | *-* | 3 | 0.51 (0.16-1.58) | - | *-* | - | *-* | - | *-* |
| *Post-vaccination interval* |  |  |  |  |  |  |  |  |  |  |  |  |
| 1-14 days | 105 | 0.78 (0.63-0.95) | 29 | 3.12 (2.03-4.78) | 64 | 0.78 (0.60-1.01) | 11 | 1.56 (0.82-2.98) | 18 | 0.78 (0.48-1.28) | 4 | 1.72 (0.59-5.05) |
| 15-28 days | 101 | 0.74 (0.60-0.91) | 23 | 1.99 (1.26-3.13) | 80 | 0.98 (0.77-1.23) | 10 | 1.31 (0.67-2.54) | 22 | 0.95 (0.61-1.49) | 5 | 1.57 (0.60-4.07) |
| 29-59 days | 277 | 0.95 (0.83-1.08) | 43 | 1.30 (0.89-1.90) | 181 | 1.02 (0.87-1.20) | 22 | 1.07 (0.64-1.77) | 49 | 0.96 (0.70-1.31) | 7 | 0.68 (0.29-1.59) |
| 60-90 days | 261 | 0.94 (0.82-1.09) | 57 | 1.48 (1.03-2.14) | 171 | 1.00 (0.83-1.20) | 31 | 1.42 (0.87-2.30) | 39 | 0.76 (0.52-1.09) | 20 | 1.50 (0.81-2.79) |
| AMI: acute myocardial infarction IRR: incident rate ratio CI: confidence interval  ^a^These are results from self-controlled case series analysis using data for first-time AMI, stroke and pulmonary embolism patients who were vaccinated with Pandemrix. The start of observation period was May 1, 2009 and end of observation period was September 30, 2010 or the day of emigration or death (whichever came first). Person-time of each vaccinated individual was divided into following risk-periods: pre-vaccination interval (15-28 days, 1-14 days prior to vaccination) and post-vaccination intervals (1-14 days, 15-28 days, 29-59 days, 60-90 days following vaccination). ^b^ All remaining part of the observation period was used for baseline comparison (unexposed person-time).  ^c^ Adjusted for calendar period (January-March, April-August, and September-December) | | | | | | | | | | | | |

| eTable6 Incident Rate Ratios, with 95% Confidence Intervals (CIs) For Different Cardiovascular Events^a^ Following Influenza Vaccination among high risk group, stratified by anti-coagulants or anti-platelet users versus non-users (anti-coagulants and anti-platelet users) | | | | | | | | | | | | |
| --- | --- | --- | --- | --- | --- | --- | --- | --- | --- | --- | --- | --- |
|  |  | **AMI**  **(n=5021)** | | |  | **Stroke**  **(n= 3134)** | | |  | **PE**  **(n=859)** | | |
|  |  | **antiplatelet or anticoagulant users**  **(n=4139)** |  | **antiplatelet & anticoagulant**  **non-users**  **(n=882)** |  | **antiplatelet or anticoagulant users**  **(n=2509)** |  | **antiplatelet & anticoagulant**  **non-users**  **(n=625)** |  | **antiplatelet or anticoagulant drug users**  **(n=667)** |  | **antiplatelet & anticoagulant**  **non-users**  **(n=192)** |
|  | **N** | **IRR (95%CI)** | **N** | **IRR (95%CI)** | **N** | **IRR (95%CI)** | **N** | **IRR (95%CI)** | **N** | **IRR (95%CI)** | **N** | **IRR (95%CI)** |
| Baseline period^b^ | 3011 | 1.0 (ref.) | 351 | 1.0 (ref.) | 1737 | 1.00 (ref.) | 330 | 1.0 (ref) | 482 | 1.00 (ref.) | 83 | 1.0 (ref) |
| *Pre vaccination interval* |  |  |  |  |  |  |  |  |  |  |  |  |
| 1-14 days | 119 | 0.87 (0.72-1.05) | 5 | 0.52 (0.25-1.06) | 51 | 0.71 (0.53-0.95) | 4 | 0.31 (0.11-0.85) | 28 | 1.30 (0.87-1.95) | 1 | 0.30 (0.04-2.24) |
| On the day of vaccination | 1 | 0.10 (0.01-0.73) | 2 | 1.81 (0.45-7.30) | 3 | 0.59 (0.19-1.83) | 0 | - | 0 | *-* |  |  |
| *Post vaccination interval* |  |  |  |  |  |  |  |  |  |  |  |  |
| 1-14 days | 62 | 0.45 (0.35-0.58) | 43 | 3.51 (2.46-5.01) | 45 | 0.63 (0.46-0.85) | 19 | 1.48 (0.91-2.42) | 11 | 0.51 (0.28-0.94) | 7 | 2.13 (0.92-4.89) |
| 15-28 days | 68 | 0.50 (0.39-0.64) | 33 | 2.65 (1.80-3.91) | 55 | 0.78 (0.59-1.03) | 25 | 1.93 (1.25-2.99) | 10 | 0.46 (0.24-0.87) | 12 | 3.59 (1.83-7.03) |
| 29-59 days | 184 | 0.63 (0.53-0.74) | 94 | 3.31 (2.52-4.35) | 134 | 0.88 (0.72-1.07) | 47 | 1.61 (1.13-2.29) | 28 | 0.56 (0.36-0.85) | 21 | 2.75 (1.55-4.85) |
| 60-90 days | 179 | 0.65 (0.53-0.81) | 79 | 2.63 (1.90-3.64) | 122 | 0.84 (0.65-1.09) | 48 | 1.59 (1.05-2.42) | 25 | 0.46 (0.26-0.80) | 14 | 1.74 (0.84-3.60) |
| 91-120 days | 191 | 0.72 (0.58-0.89) | 100 | 3.36 (2.50-4.50) | 113 | 0.80 (0.62-1.03) | 54 | 1.84 (1.24-2.72) | 34 | 0.65 (0.39-1.09) | 15 | 1.91 (0.95-3.84) |
| 121-180 days | 324 | 0.60 (0.53-0.68) | 175 | 2.62 (2.17-3.18) | 249 | 0.80 (0.69-0.92) | 98 | 1.58 (1.24-2.01) | 49 | 0.52 (0.38-0.72) | 39 | 2.46 (1.64-3.70) |
| IRR: incident rate ratio  CI: confidence interval  ^a^ All Other Parts of The Observation Period  ^b^ Adjusted For Calendar Period (January-March, April-August, And September-December) | | | | | | | | | | | | |
